# Supplementary material for: AaHog1 Regulates Infective Structural Differentiation Mediated by Physicochemical Signals from Pear Fruit Cuticular Wax, Stress Response, and Alternaria alternata Pathogenicity
Source: J Fungi (Basel). 2022 Mar 6;8(3):266. doi: 10.3390/jof8030266 (PMC8952436; doi:10.3390/jof8030266)
Supplement: Supplementary file 1 [file jof-08-00266-s001.zip › jof-1603117-supplementary.pdf]

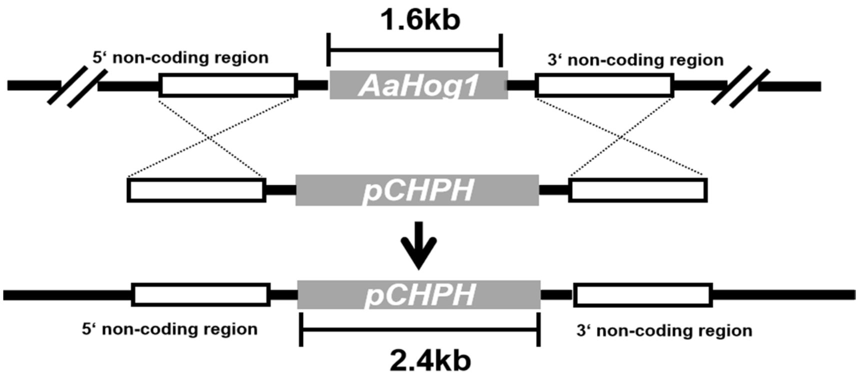

Supplementary Figure S1. Homologous recombination strategy.

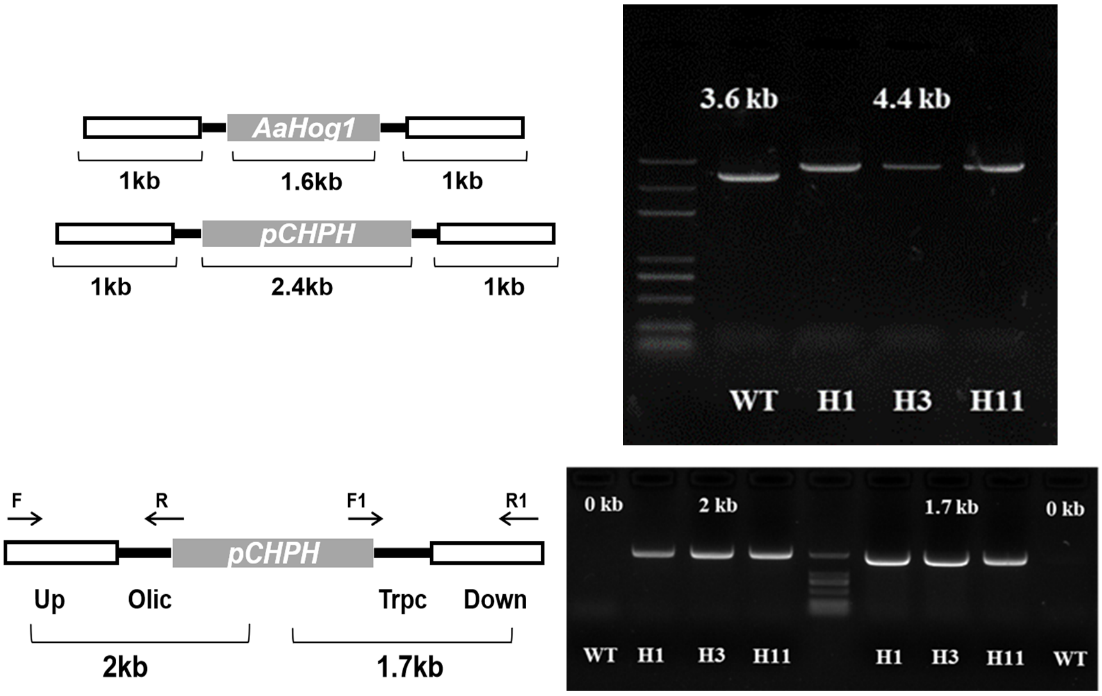

Supplementary Figure S2. Validation of transformants.

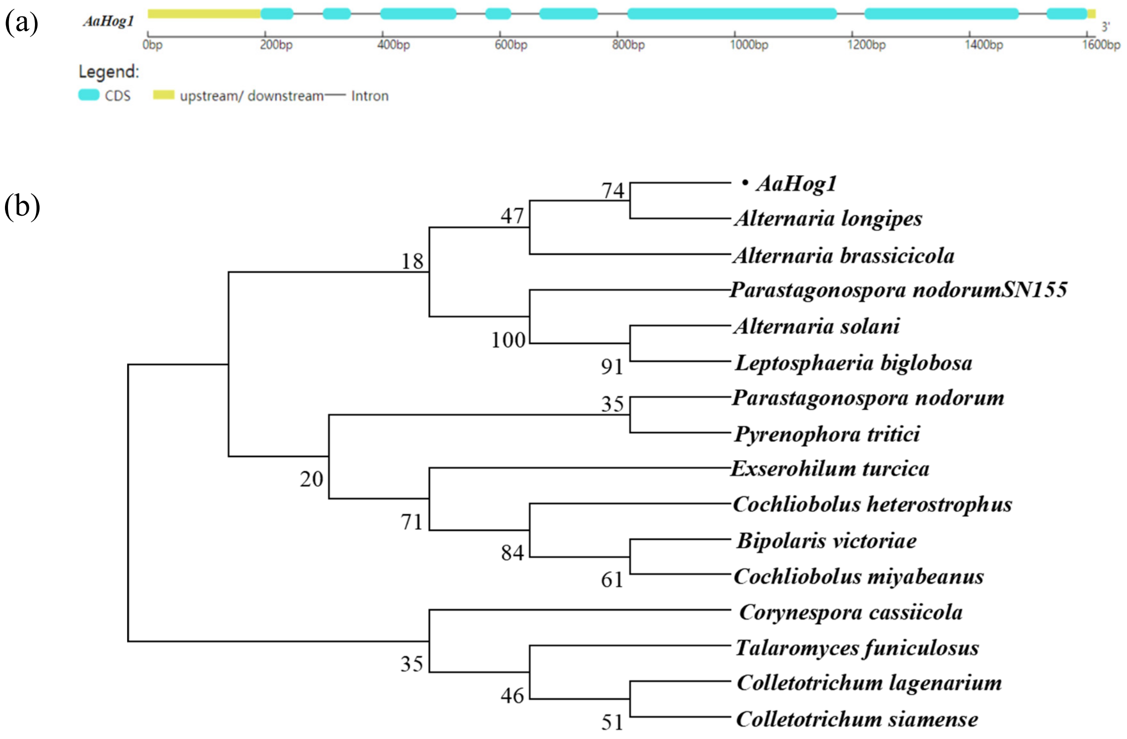

Supplementary Figure S3. Characterization and identification of the *AaHog1* in *A. alternata.*

Supplementary Table S1. Primers used to amplify up and down sequences of target genes.

Gene	Primer sequences (5' - 3')	Target function
AaHog1-up	F: ACAGCTATGACCATGATTACGAATTCGATAGCCGCAGCATACCATT<br>R: GATCCCCGGGTACCGAGCTCGAATTCGCTCCGAGGGATCTTTCTTGT	5' sequence amplification of *AaHog1*
AaHog1-down	F: CATGCATGGTTGCCTAACTCGGCGCGCCTTGATGCGGCGGCACAGGAG<br>R: GACGGCCAGTGCCAAGCTTCGGCGCGCCCGGCGGAGGGTAAAGTAGGA	3' sequence amplification of *AaHog1*
AaHog1-V	F: TCGGGTGGGTTACAACAGAA<br>R: GTCCCTCCTGGTTCTTTAGC	Validation of transformants
**Olic/trpc**	F: TAGAGTAGATGCCGACCGG<br>R: CTGAAAGCACGAGATTCTTC	Validation of transformants
AaHog1-c	F: GCATGGACGAGCTGTACAAGGAGCTCATGGCGGAATTCGTACGCGC<br>R: ATGGAGCTATTAAATCACTATCTAGATTAGCTGCCGTTGTTCTCTT	Target gene amplification of *AaHog1-c*
**AaHog1-c-V**	F: ATGGCGGAATTCGTACGC<br>R: TTAGCTGCCGTTGTTCTCTT	Validation of transformants
AaHog1-qPCR	F: GACCCCACTGATGAGCCGATTG<br>R: GCCGCATCAACATTGTGGTAGTCC	Quantitative analysis of *AaHog1*
